# Socio-Economic Status and Health: Evaluation of Human Biomonitored Chemical Exposure to Per- and Polyfluorinated Substances across Status

**DOI:** 10.3390/ijerph15122818

**Published:** 2018-12-11

**Authors:** Jurgen Buekers, Ann Colles, Christa Cornelis, Bert Morrens, Eva Govarts, Greet Schoeters

**Affiliations:** 1 Flemish Institute for Technological Research (VITO)—Sustainable Health, 2400 Mol, Belgium; ann.colles@vito.be (A.C.); Christa.cornelis@vito.be (C.C.); Eva.govarts@vito.be (E.G.); Greet.schoeters@vito.be (G.S.); 2 Sociology Department, University of Antwerp (UA), 2000 Antwerpen, Belgium; bert.morrens@uantwerpen.be

**Keywords:** PFAS, SES, socio-economic status, income, education, health inequity, human biomonitoring, HBM, HBM4EU

## Abstract

Research on the environment, health, and well-being nexus (EHWB) is shifting from a silo toward a systemic approach that includes the socio-economic context. To disentangle further the complex interplay between the socio-exposome and internal chemical exposure, we performed a meta-analysis of human biomonitoring (HBM) studies with internal exposure data on per-and polyfluoroalkyl substances (PFASs) and detailed information on risk factors, including descriptors of socio-economic status (SES) of the study population. PFASs are persistent in nature, and some have endocrine-disrupting properties. Individual studies have shown that HBM biomarker concentrations of PFASs generally increase with SES indicators, e.g., for income. Based on a meta-analysis (five studies) of the associations between PFASs and SES indicators, the magnitude of the association could be estimated. For the SES indicator income, changes in income were expressed by a factor change, which was corrected by the Gini coefficient to take into account the differences in income categories between studies, and the income range between countries. For the SES indicator education, we had to conclude that descriptors (<college, x years of study, etc.) differed too widely between studies to perform a meta-analysis. Therefore, the use of the uniform ISCED (International Standard Classification of Education) is recommended in future studies. The meta-analysis showed that a higher income is associated with a higher internal exposure to PFASs (PFOS or perfluorooctanesulfonic acid, PFOA or perfluorooctanoic acid, PFNA or perfluorononanoic acid, PFHxS or perfluorohexane sulfonate). This is opposite to the environmental justice hypothesis, referring to an inequitable distribution of detrimental environmental effects toward poor and minority communities by a practice or policy. With a doubling of the income, internal exposure increased on average by 10%–14%. Possible explanations for this difference are given, e.g., underlying differences in diet. However, other sources can also contribute, and the exact causes of SES-related differences in PFAS concentrations remain unclear. Studies are needed that include social descriptors together with lifestyle and dietary information as explanatory variables for internal chemical exposure levels. This will help clarify the underlying factors that link SES with inequity to environmental exposures, and will raise awareness and knowledge to strengthen the capacities of people and communities to advocate chemical exposure reduction in order to reduce this health inequity.

## 1. Introduction

Policymakers have already recognized for decades that human health and well-being are intricately linked to environmental quality and most recently it appears as a cornerstone in the European Union (EU)’s seventh Environment Action Plan [1]. The policy focus is now shifting from studying single environmental pollution (silo approach) toward systemic challenges in which persuasive health inequality and the association with social and economic factors are also considered [2,3,4,5]. At the Sixth Ministerial Conference on Environment and Health in Ostrava in 2017, organized by the WHO (World Health Organization) European Region, it was stated that “… exposure to harmful chemicals and the destabilization of ecosystems threaten the right to health, and disproportionately affect social disadvantaged and vulnerable populations groups, thereby exacerbating inequalities”. Therefore, equity, social inclusion, and gender equality need to be considered in policies [6]. Further, one of the ambitions of the EU2020 strategies for a healthier population is the reduction of health inequality in an aging Europe [7]. Fragmented evidence shows that deprived, minority, and low-income populations often live in areas with higher environmental pollution and limited access to green space, and lack financial and educational capacities to avoid relatively higher exposure to environmental stressors [8,9,10]. This evidence is often framed in term of environmental (in)justice or environmental inequity, which focus on the unequal and unfair distribution of environmental hazards and access to environmental decision-making processes [11]. Environmental inequity is not a homogenous construct, but its manifestation can differ between subpopulations, local areas, and countries that might be considered fairly similar [12]. Through participative processes and trust building, groups of different socio-economic and ethnic backgrounds can be reached for environmental health research [13] and environmental health promotion [14,15]. However, knowledge gaps still exist on how social and economic determinants are exactly linked with the environment, health, and well-being nexus [16,17].

**Figure 1:**
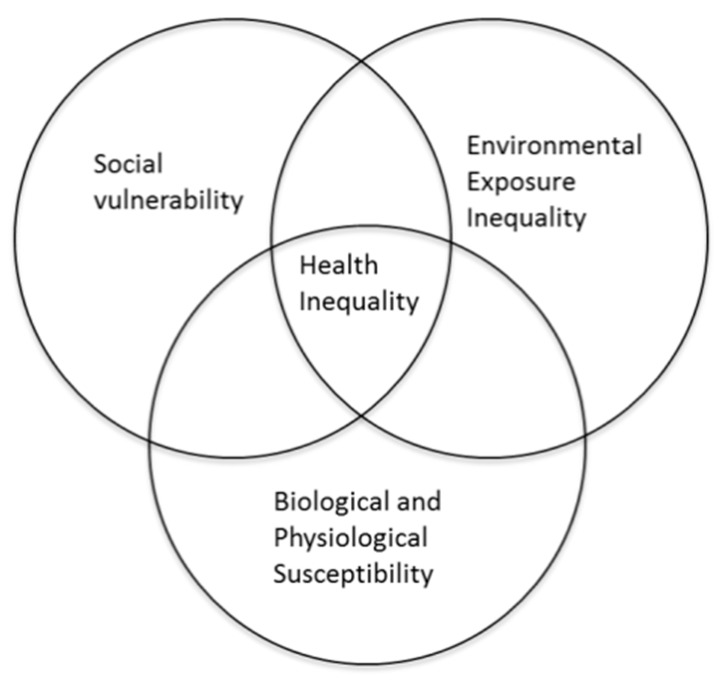
Interaction between health inequalities, environmental exposure inequality, different biological and physiological susceptibility and social vulnerability. Figure based on study of Morello-Frosch et al. [22] and Frumkin [23].

In general, indicators of socio-economic status (SES) are meant to give information on the individual’s access to social and economic resources. Common indicators for SES based on surveys are education, income, and occupation. In relation to health effects, these indicators are not interchangeable. Various SES indicators may capture different aspects of the overall health impact [18]. Socio-economic indicators have been shown to modify or confound exposure-response associations. Many exposure-response associations are adjusted for socio-economic variables [19,20]. More recently, studies show that socio-economic factors influence exposure and specific health outcomes, while the underlying causal factors are largely unknown [21]. Social and environmental stressors can combine additively (or synergistically) to produce health inequalities [22,23,24]. Together, these stressors may lead to a cumulative impact. Concepts supporting the principle of a cumulative impact are: a) inequality in exposure to environmental hazards, b) differences in biological and physiological susceptibility, and c) amplification by social vulnerability factors (access to healthcare, education about prevention, etc.). Also stress may have an additional influence on health outcomes [25]. A combination of all may lead to health inequalities (Figure 1) or health inequities between socially defined groups, as these are unfair, unjust and avoidable. Next to the evidence that SES is related to multiple risk factors, there is evidence that SES influences multiple diseases, that the deployment of resources plays a critical role in the association between SES and health status, and that the association is reproduced over time [24]. Still, it’s not completely clear how environmental risk factors operate in the reality of the social environment and vice versa. At the research level, a new conceptual framework for integrative environmental health research is the socio-exposome proposed by Senier et al. [26]. With the term exposome, researchers want to quantify internal and external exposure as precisely as gene expression measurements. The term socio-exposome requires more comprehensive data on HBM, environmental exposures, together with information on socio-economic conditions and inequalities.

Tools exist to survey substantial differences in population exposure and health. One of these is HBM or human biomonitoring, by which individual differences in internal exposure (exposure biomarkers) and effect (effect biomarkers) can be assessed by chemical analysis of pollutants, their metabolites, or biological responses in human fluids or tissues. Most HBM studies collect also personal data on lifestyle and dietary habits, health, and some indicators for SES such as education, income, or occupation. HBM allows tracking flows of chemicals through society. Such integrated assessment can support the design of preventative environmental health policies to identify vulnerable communities, and reduce the health impact, health inequality, and related healthcare costs [27]. Data to quantify the environmental health inequality situation are not abundant. Recently, the HBM4EU project (human biomonitoring for Europe) started. It is a Horizon2020 Framework Project for the development of a sustainable European-wide HBM network (2017–2021) [28]. Under HBM4EU, all partners are requested to send their HBM data for substances and groups of substances to the European Environment Agency (EEA) for inclusion in IPCHEM: the Information Platform for Chemical Monitoring [29]. Initially, IPCHEM will include metadata and mostly aggregated data of past and ongoing HBM studies in Europe. Next to variables as e.g., age, sex, and location linked to the HBM data, also data on education as indicator of SES will be gathered. Integrating data from the health, environment, social, and economic domains is a major challenge that depends on the available datasets and underpinning data infrastructure.

Individual studies show associations between the exposure to per- and polyfluoroalkyl substances (PFASs) through HBM and SES indicators such as income and education [30,31]. Our goal in this study was to investigate whether a meta-analysis could be done for the association between SES indicators income and education and HBM data, based on data of different countries, and if an estimate of the magnitude of the association could be given. Secondly, we wanted to elaborate on the main identified exposure determinants influencing the exposure biomarker SES relationship. Can differences in diet play a role? PFASs are synthetic chemicals that are widely used in the manufacturing of industrial and consumer products such as food packaging, and stain-resistant coatings, water and oil-repellent coatings for textiles and paper since their introduction in the 1950s. Some examples are PFOS (perfluorooctanesulfonic acid), PFOA (perfluorooctanoic acid), PFNA (perfluorononanoic acid) and PFHxS (perfluorohexane sulfonate). PFASs are persistent in the environment [32]. PFOS is included in the list of the Stockholm Convention (2009) on persistent organic chemicals; PFOA and PFHXS are recommended to be added to the list. PFOS was phased out by the major manufacturers between 2000–2002. The restricted use of PFOS was implemented in Europe from June 2008. Since 2000, there has been a clear decline in blood levels of PFOS and a minor decline for PFOA [33,34,35,36]. PFOS and PFOA are oleophobic, and accumulation in fat is therefore unlikely. They bind to serum albumin and build up in protein-rich compartments of the body as liver and kidney [37]. Mean human half-lives of serum elimination were estimated in the study of Olsen et al. [38]: 5.4 years for PFOS and 3.8 years for PFOA. PFAS are linked to endocrine disruption (ED), liver, developmental and immune toxicity [39,40,41]. In the Flemish Environment and Health Studies (FLEHS), significant associations were found between adult plasma concentrations of PFASs and SES (income, education) (see further). General population exposure mainly takes place through ingestion and additionally inhalation [42,43]. In some cases, the indoor environment (dust and air) may contribute significantly to total body burden [42].

## 2. Methodology

### 2.1. Literature Search

Studies providing both data on PFAS concentrations in the serum or plasma of adolescents and adults (no cord blood) and on socio-economic status were the primary target. A literature search was done using PubMed as search engine. In Table 1, an outline of the search strategy is given. Based on titles and abstract content, 11 studies were selected for more detailed analysis. A main requisite was that based on the abstract, it should be obvious that HBM data are included in the selected study. Also, references in the selected studies, which might be relevant for this analysis, were checked in detail [35,44,45,46,47]. A restriction was set to studies from the United States (US), Canada, and Europe, which have a long history in HBM and a comparable timeframe of historical industrial revolution. This list also includes the study of Colles et al. [48] describing Flemish data on the levels of PFASs and determinants of exposure. The number of studies reporting on the internal chemical exposure to PFASs and socioeconomic factors is also limited when other search engines such as Web of Knowledge were used.

### 2.2. Meta-Analysis

In the international literature, HBM biomarkers of exposure are often associated with SES indicators such as income or educational level. Generally, in regression models of HBM studies, the percent change of HBM biomarker concentrations is given per income category and/or educational level. For income, three to four categories that are more or less uniformly divided across the income range are mostly used e.g., Kato et al. [57] reported serum PFAS concentrations for the quartile income categories <$20,000, $20,000–40,000, $40,000–80,000, and >$80,000. To compare the percent change in biomarker concentrations per change of income between studies, the income change was expressed in terms of factors (ratios with lowest category as denominator). In this case, for the study of Kato et al., the maximum factor by which income changes was set to four (= $80,000/$20,000). To go further and make the comparison between countries, this factor was corrected for income inequality between countries by dividing the change in income factor by the Gini coefficient for income inequality (data retrieved from the OECD, or Organization for Economic Cooperation and Development; year 2013; scale 0–1; USA: 0.40, Belgium: 0.27, Norway: 0.25) [58]. Hereby, the assumption is made that for HBM studies focusing on a metropolitan area, an identical Gini coefficient can be used as derived at country level by the OECD.

The mean percentage changes (±95% confidence interval or CI) in PFAS concentrations as a function of factor change in income, derived from (multiple) linear regression models, were combined for a number of studies (oldest study dating back to 2012). In most studies, the highest income category was chosen as a reference for comparing the change in biomarker concentration, while in some this was the lowest income category. To be consistent, these were all corrected toward the highest income category. A meta-analysis studying the relationship between factor change in income corrected by the Gini coefficient and human PFAS concentrations was carried out. To take into account the information embedded in the fault on the change in PFAS concentrations provided in the individual studies (95% CI), a weighted least squares (WLS) (Excel 2010 (Microsoft Corporation, Redmond, WA, USA) (add-in: Real-Statistics)) linear regression model was applied. The weights were set equal to the inverse of the standard deviations on the percent change in PFAS concentrations. PFASs reported in the studies were usually PFOS, PFOA, PFNA, and PFHxS.

For education, a combination of the individual results was not recommended here (see Results and Discussion).

### 2.3. Determining Factors

Different factors may underlie the association between biomarker PFAS concentrations and SES indicators. We made a short list of the possibilities that have been mentioned in the individual studies. Further, we explored in an extra calculation for PFOS exposure in Belgium whether differences in diet between groups of different SES may explain findings in HBM studies.

## 3. Results and Discussion

A detailed stratification of the data (different SES categories and associated biomarker concentrations) is essential to compare results between studies. Some of the studies selected in Table 1, and the references therein, reported on PFAS concentrations without focusing in detail on SES data [44,52,53,54,55]. These studies are not considered further. Also, studies reporting on PFAS concentrations in cord blood (not maternal serum or plasma) are not taken up in our review, although placental transfer in humans has been confirmed [45]. Briefly, in the study of Apelberg et al. [53], higher cord blood concentrations of PFASs (PFOS and PFOA) were observed with higher educational levels of women in Baltimore, Maryland, although the association was not significant (*p* > 0.05). In the study of Arbuckle et al. [46], umbilical cord blood levels of PFASs were measured in Canadian mothers. In a univariate analysis, household income was significantly negatively associated with PFOS concentrations (decreasing PFOS concentration with increasing income; 9.1 ng/mL to 5.5 ng/mL; *p* = 0.01), and maternal education was significantly positive associated with PFOA concentrations (increasing PFOA concentration with increasing education; 0.1 to 1.3 ng/mL; *p* = 0.03). In the final stepwise multiple regression model, both income and education were not retained as significant variables (*p* > 0.05).

### 3.1. Income and HBM

In the Canadian MIREC (Maternal–Infant Research on Environmental Chemicals) study reported in Table 1, a significantly positive association was found between maternal PFOS, PFOA concentrations, and household income. The association with PFHxS was not significant [51,56]. Overall, this study was in line with findings in the international literature. The study was not considered further, as the level of detail of the data was not high enough for inclusion in our analysis (no detailed results of percent change in PFAS concentrations or regression coefficient β from logistic regression model per income category). The study of Jain [47] described findings of the National Health and Nutrition Examination Survey, NHANES 2003–2008. Results of the association with socio-economic status were similar to the study of Nelson et al. [30] (NHANES 2003–2006) focusing on social disparities in exposure. To avoid double counting in our analysis, the results of the study of Jain were not included.

The results of five studies with regression analysis of income categories versus the corresponding PFAS concentrations in blood could be combined (Table 2). For all of these studies, the percent change per income category was available. Comparing absolute concentrations of PFASs between studies is not straightforward, given the differences in analytical techniques, age, time period, etc. Only human biomonitoring programs with harmonized procedures for recruiting participants, chemical analysis, and data treatment allow the robust detection of variations in chemical body burden. The comparison of relative units such as percentages, factor changes, etc. can be done across studies.

Income categories are more or less uniformly distributed across the income range, and in most studies, PFAS concentrations are reported for three to four income categories. Factor changes in income were calculated for the five remaining studies. The largest uncertainty in this calculation was for the study of Brantsæter et al. [35]: both parents <300,000 Norwegian Krone (NOK), one ≥300,000 NOK, and both ≥300,000 NOK. The factor change was set here to two for income changes between the lowest and highest categories. Other studies reported more into detail on the income categories.

**Figure 2:**
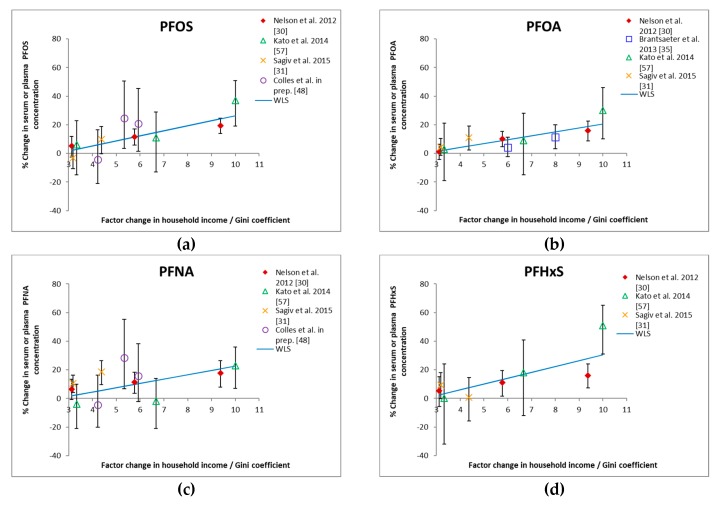
Association between factor change in household income/Gini coefficient and percent change in adult PFAS concentrations in plasma or serum (**a**: PFOS, **b**: PFOA, **c**: PFNA and **d**: PFHxS). Factor change calculated from uniform distribution of household incomes over three to four income categories with lowest income category as denominator. Gini coefficient retrieved from the Organization for Economic Cooperation and Development (OECD) (scale 0–1; USA: 0.40, Belgium: 0.27, Norway: 0.25). A change on the X-axis of 10 for the US study of Kato et al. [57] means an increase in income factor of 10 × 0.40 = 4, which corresponds with an increase in percentage in PFOS concentration of 37%. Error bars indicate 95% CI. The straight line is based on a weighted least squares (WLS) with weights equal to the inverse of the standard deviation on the percent changes in PFAS concentrations and intersection with the X-axis equal to (2.5,0). Slopes were 3.5 (*p* < 0.001) for PFOS, 2.7 for PFOA (*p* < 0.001), 3.0 for PFNA (0.01 > *p* > 0.001) and 4.1 for PFHxS (0.05 > *p* > 0.01).

The studies of Nelson et al. [30], Kato et al. [57], and Sagiv et al. [31] found a significant positive change in human concentrations of PFOS, PFOA, PFNA, and PFHxS with increasing income. The Norwegian study of Brantsæter et al. [35] found a significant positive change in PFOA concentrations with increasing income. In their study, income and education were not included in the same regression model, and for PFOS, PFNA, and PFHxS, there was a significant positive association with education. The study of Colles et al. [48] found a significant positive change in PFOS and PFNA concentrations with increasing income. When all of the studies are combined (Figure 2), a significant (*p* < 0.05) increase in PFAS concentrations was found with increasing income. The weighted least squares analysis showed that the serum PFAS concentration increased on average 10%–14% for a doubling of the income in a country with a Gini coefficient equal to 0.33. The value of 0.33 was chosen as the middle between the range of 0.40 (Gini coefficient of the US) and 0.25 (Gini coefficient of Norway).

### 3.2. Education and HBM

Education may impact exposure via the ability to access and interpret health-related information [59]. When studying the descriptor for education (x years of study, <college, etc.), this differed too strongly between individual studies, making a meta-analysis impossible. The association between educational level and PFAS biomarker concentrations found in the individual studies is discussed shortly in the next paragraph.

In seven studies, the association between PFAS biomarker concentrations and education was analyzed. The study of Lauritzen et al. [49], analyzing perfluoroalkyl substances in the maternal serum of women in Norway and Sweden, reported on an increase of PFOS and PFOA concentrations with educational level, but this was not significant. The study of Sochorová et al. [50] on the PFASs in Czech adults reported an increase in concentrations of PFOA, PFNA, PFHxS, and PFOS with educational level, which were not (except for PFOA) to borderline significant after correction for co-variables. The Canadian MIREC study showed that for education, a significantly negative association was found between lower maternal education and PFOS concentrations (*p* < 0.05), while for PFOA and PFHxS, associations were not significant [51]. The study of Nelson et al. [30] found lower human PFOS, PFOA, PFNA, and PFHxS concentrations with lower educational level; however, changes were insignificant. Colles et al. [48] reported on significantly higher PFOA concentrations with higher educational level in (Flemish) Belgian adults (21% increase). The concentration of PFOS increased 23% with educational level, but this was borderline insignificant. In the study of Brantsæter et al. [35], a significant increase of PFOS, PFNA, and PFHxS biomarker concentrations in pregnant women was found with educational level. In contrast, the study of Sagiv et al. [31] found higher concentrations of PFOS, PFOA, and PFHxS at lower educational levels. The trend was opposite with the one for income in the study of Sagiv et al. [31]. Thus, patterns were here not uniform across SES indicators. The authors were unable to explain this inconsistency between education and income. The educational level in the study was defined roughly: <college, college, >college, which may make it harder to capture SES differences.

As already mentioned, the descriptor for education differed strongly between studies reporting on PFAS. In the DEMO(COPHES) (Demonstration Of A Study To Coordinate And Perform Human Biomonitoring On A European Scale) project [60], in which a common approach for HBM surveys in the European Union (EU) was developed and tested, the uniform International Standard Classification of Education (ISCED) was used as a descriptor, and enabled making data on educational level comparable over time and across EU countries [61]. The ISCED is the reference international classification for organizing education programs and related qualifications by levels and fields. Its use is highly recommended for further studies.

### 3.3. PIR (Poverty Income Ratio) and HBM

PIR is defined as the ratio of the family’s self-reported income to the family’s appropriate poverty threshold [62]. In some studies, PIR was used as an indicator for SES; however, income and education remain the indicators that are most often used. For example, the study of Tyrrell et al. [21] reported on the association between adult PFAS concentrations and PIR for the NHANES campaigns covering the years 2001–2010: PFOA and PFNA were significantly positive associated with PIR.

### 3.4. Determining Factors

Since income and education may be closely associated with exposure, detailed data stratification could provide clues about the particular aspects of life that are most closely associated with the magnitude of exposure and with the socio-economic status of the study subjects [63]. This means that for aggregated data in a database, sufficient and quantitatively well-defined categories should be available to capture differences in outcome or see gradients. This study did not identify in detail the underlying determinants of the association between SES and adolescent and adult PFAS concentrations, but it builds further on the reasons given in the studies included in the meta-analysis and the references therein. A short list of possibilities (not exhaustive and sequence irrelevant) mentioned in the individual studies is given in the next paragraphs.

Differences in diet between income groups exists. In Europe, individuals of higher SES tend to consume more fish, marine food, vegetables, and fruit, which are potential sources of PFASs, compared to individuals of lower SES [43,64,65]. When the effect of income, education, and occupation on diet quality are examined, studies have often shown that income and education are important predictors of diet quality [66]. Overall, there is no consensus if the choice in diet quality is mainly driven by income or education. More and more studies have argued that SES is a complex concept with different associations with exposure [67,68].

The continuous (linear) increase in adult PFAS concentrations by income, certainly in the US studies [30,57] (see Figure 2), remains to be further investigated. For PFOS, diet is seen as the most important contributor to the PFOS body burden [43]. Therefore, could the continuous increase in PFOS biomarker concentration by income be related to the continuous change in consumption patterns of major foodstuffs by different SES groups? Differences in food consumption patterns between groups of different income and education levels are observed. For Belgian adults, for which we have data available, there is a continuous increase in the consumption of fish, vegetables, and fruit with increasing education (see Table 3). For potatoes and meat, an opposite trend is observed. 

A rough estimate on PFOS intake through diet in Belgian adults by SES shows further that fish and fruit are major contributors to the total dietary PFOS intake (see Table 4; calculation based on food PFOS concentrations and food consumption (Table 3)). In the calculation shown in Table 4, assumed PFOS concentrations in foodstuffs were retrieved from the recent EU PERFOOD (PERFluorinated Organics in Our Diet) project [69], and were set equal to 0.003 ng/g for vegetables [70], 0.043 ng/g for meat [71], 0.061 ng/g for eggs [71], 0.098 ng/g for fruit [72] (mean of positive detects), 0.301 ng/g for freshwater/marine fish [71], and 0.523 ng/g for seafood [71]. The consumption of fish versus seafood was set at a ratio of 1.5. Concentrations of PFOS were below the limit of quantification for potatoes [70]. The study of Cornelis et al. [43] and the European Food Safety Authority (EFSA) report [73] on PFOS & PFOA in the food chain showed a significantly higher contamination of food from animal origin with PFASs. Also, in the study of Cornelis et al. [43], potatoes and fruit contained significant amounts of PFOS. For fruit, the PFOS level was 0.350 ng/g. The total average PFOS intake in the example, as worked out here, was around 370 pg/kg BW/day, which is in agreement with the estimate in the study of Klenow et al. [74].

Table 4 indicates that the total variation between the two groups with most pronounced difference in education is equal to +22% for total PFOS intake with the lowest educational group as reference, +17% for both fish and seafood intake, −18% for meat intake, and +63% for fruit intake. The intake of fish, seafood, and fruit have an opposite effect on the increased intake of PFOS by education compared to the intake of meat. People with higher education in Belgium tend to consume less meat than people with a lower education. Surprisingly, the increase of +22% intake of PFOS by diet for groups of higher educational level that was calculated here corresponds reasonably well with findings in the Flemish HBM study (FLEHS 2012–2015: around 20%). In Belgium, the average gross income/person of individuals in ISCED categories 0–2, 3–6, and 7–8 equals respectively €2756, €3617, and €5236 (Euro 2015) [76]. Thus, income differences between the lowest and highest educated people in Belgium is almost a factor 2. Although income and education are not completely interchangeable for dietary behaviour, we see that on average, an increase in income by a factor of two can explain a 22% higher intake of PFOS in the high SES group. When we compare this value with data for income found in the Flemish study of Colles et al. [48], it was found that the change of adult PFOS serum concentrations by different income groups is about 30% (see Table 2); this figure is larger than the calculated 22% here, although income levels may differ. Based on this rough calculation, it is clear that diet alone can explain a large part of the observed variation of PFOS serum concentrations by different educational and income groups in Belgium. Based on Table 4, it is also clear that in Belgium, fish and seafood are an important food category for the dietary exposure to PFOS for adults, but fruit is as well. The contribution of vegetables (2%) and eggs (2%) to the total PFOS intake was minor. However, there are several points of attention, and this calculation should be nuanced: e.g., PFOS food concentrations differ across food studies; thus, the uncertainty is large. Different ways to handle samples below the limit of quantification create variability. Differences in food quantity between groups of different SES will play a role, but diet quality does as well. The individuals for which we have food data by SES differ from the ones for which we have HBM data. The food consumption study was for the Belgium population, while the HBM results here are Flemish. This ‘back of the envelope’ calculation was only done for PFOS in Belgium, and more detailed data on income, education, and HBM biomarkers are needed for a more precise estimate. The situation may also differ for other countries. The full picture for the underlying causes of SES-linked differences in PFAS concentrations needs further elucidation.

As in Europe, fish and seafood consumption are major sources of PFOS intake in the US (United States of America) and Canada; the consumption of meat was estimated as the greatest source of perfluorocarboxylates and PFOS [77,78]. The concentration of PFOS in a Canadian food study was equal to 2.7 ng/g (beef steak; [77]) and the intake of beef steak, roast beef, and grounded beef accounted for 90% of the average daily dietary PFOS intake for Canadians >12 years old. However, the Canadian food intake values that were used dated from 1972 [77]. In Canada and the US, there is a strong positive relationship between the level of household income and beef demand. The income elasticity of beef demand is estimated at 0.54 in Canada. This means that a 1% increase in income results in a 0.54% increase in demand for beef [79]. In the US, the estimated income elasticity varies in different studies, with a positive range between 0.4 [80] and 0.9 [81]. When looking at the increase in income in the studies of Nelson et al. (2012) and Kato et al. (2014), the income changes by a factor of four, which means that beef demand can change drastically between different income groups. However, meat consumption is likely not the only food source influencing income-related human PFAS concentrations in the US and Canada. The study of Tyrell et al. [21], focusing on five NHANES waves (2001–2010), found that the relationship between the poverty income ratio (PIR) and PFNA was mediated to some extent by shellfish consumption. Fish consumption was not noted to be a mediator.

Exposure to PFAS through food can have an environmental source, but food contact materials can also contribute. The PFASs used in these materials, such as paper plates, food wrappers, etc., have been shown to migrate in food [82,83]. From 1970 onwards, phosphate esters based on perfluorooctanesulfonamido ethanol (N-EtFOSE) were used in food contact materials. These esters break down to N-EtFOSE, which can further break down to PFOS [84,85]. The association of PFASs in food contact materials and SES is currently unclear.

A second source of PFASs exposure, next to diet, is exposure through contact with consumer products. Households with higher income can purchase expensive textiles and sport equipment containing PFASs. PFASs tend to be found in expensive fabrics that provide water proofing [86]. However, the PFAS content in consumer products and possible emissions and intake remain unclear.

Another route through which exposure may take place is inhalation. Fraser et al. [87] showed that fluorotelomer alcohols (PFAS precursors) in the air of offices contribute substantially to the body burden of PFOA and PFNA. These volatile alcohols may originate from carpets, furniture, and/or paints. Individuals of higher SES are more likely to have offices jobs. It is not clear to what extent the inhalation of PFAS precursors play a role in the total exposure picture. To conclude, some determinants may suggest why an association between PFAS biomarker concentrations and SES is found; however, the exact cause remains currently unknown.

## 4. Conclusions

The unfair distribution of internal chemical pollution across social groups remains an important social and policy issue. Different strategies try to tackle this problem (EU2020 strategy, seventh EAP, WHO Parma and Ostrava Declaration). In this study, the association between PFAS biomarker levels measured in blood samples of the general population and SES categories was studied by a meta-analysis including five studies. Additionally, determinants of exposure in relation to income and educational levels were checked as potentially underlying causes for the association between SES constructs and PFAS blood levels. The meta-analysis confirmed that socio-economic status defined by income is an important determinant of PFAS blood levels. For the SES indicator income, study-specific applied income categories differ. On top, income ranges differ between countries. By applying for each study a factor change in income between income categories and correcting this by the Gini coefficient, studies could be combined, and the magnitude of the association between the change in income and HBM PFAS concentrations could be studied. A requisite is that the level of detail given in individual studies is sufficient. In cases of income as an SES indicator, the level of detail means that PFAS concentrations (and/or modeled percentage changes) are necessary per income category, and that sufficient and quantitatively well-defined categories are available to capture differences in exposure. Databases that collect HBM data such as the IPCHEM should be structured to include exposure levels per income categories and educational levels to follow trends in exposure inequality that relate to social status. Besides this, harmonized protocols to define SES variables such as the uniform International Standard Classification of Education (ISCED) should be used if reporting. Currently, we could not perform a meta-analysis for the SES indicator education, given that applied descriptors (<college, x number years, etc.) differed too widely between studies. The meta-analysis for income showed consistently that a higher income is associated with higher PFAS concentrations, or that a low SES is not always associated with an increased chemical burden. The environmental justice hypothesis, in which persons of lower SES groups have a larger internal chemical exposure, does not hold for all of the chemicals. When for a studied population, household income is approximately distributed uniformly across three to four categories, a significant (*p* < 0.05) increase in adult PFAS concentration is identified for PFOS, PFOA, PFNA, and PFHxS. The change was similar for the studied PFASs, and the average increase in PFAS concentrations varied between 10%–14% for a doubling of the income. However, underlying factors causing this increase probably differ between the US and Europe, for which studies were included in the meta-analysis. For example, for PFOS, diet is seen at the most important factor contributing to the human body burden in both continents, but as in Europe, the consumption of fish is probably a main culprit, while in the US, beef consumption probably plays a major role. An extra calculation made for Belgium showed that differences in diet can explain a large part of the PFOS biomarker variability across SES. Either way, differences in diet are not the only underlying cause explaining the differences in human PFAS concentrations by SES category. The contribution of exposure through dermal contact with consumer products and through the inhalation of contaminated air remains unclear. Studies on the underlying causes resulting in observed SES differences of internal chemical exposure are definitely needed to unravel the socio-economic–environment–health relationship and decrease human health inequality. With that knowledge, awareness can be raised, and people can be stimulated in making smart choices reducing their exposure to chemicals [88]. Regarding the use of SES indicators income and education, both have their pros and cons, and often they capture different aspects of the health impact. Our study is not in favour of one or the other, but more consistency should be applied to compare results (e.g., application of ISCED). The variable income is sometimes seen as sensitive information to share, and is not always reported in questionnaires.

## Figures and Tables

**Table 1:** Results of literature search strategy in PubMed (performed January 2018; no restriction set on the search time period). PFAS: per-and polyfluoroalkyl substances, SES: socio-economic status.

Search Term 1 in All Fields	Search Term 2 in All Fields	Number of Studies Found	Number of Studies Selected Based on Title and Abstract Relevance	Selected Studies ^a,b^
PFAS	/	504	0	Too many results. Search term added.
	SES	2	0	/
	Socio-economic ^c^	10	1	[31]
	Education	35	3	[49,50,51]
	Income	4	0	/
	Predictor	7	1	[52]
Polyfluoroalkyl	/	265	0	Too many results. Search term added.
	SES	0	0	/
	Socio-economic	3	2	[30,53]
	Education	13	2	[54,55]
	Income	1	0	/
	Predictor	3	0	/
Perfluoroalkyl	/	1453	0	Too many results. Search term added.
	SES	2	1	[56]
	Socio-economic	6	0	/
	Education	66	0	/
	Income	7	1	[57]
	Predictor	5	0	/
Total selected			11	

^a^: Search was performed according to this table, with search sequence according to the order in this table (from top to bottom). A study was only selected once, although it could be found by different search combinations. For example: The study of Nelson et al. [30] was not only found by the search terms “polyfluoroalkyl” and “socio-economic” but also by the search terms “polyfluoroalkyl” and “income”, although it was only selected once. ^b^: also references in the selected studies were studied based on relevance of title and abstract. ^c^: socio-economic as well as socioeconomic were searched for.

**Table 2:** Overview of studies with associations between household income and adult PFAS concentrations in serum or plasma.

Reference (Sampling Time)	Country	Age Category (Years)	Size	Household Income	GM or Median Concentration (ng/mL)				% Change per Income Category from Regression Models				Remark
					PFOS	PFOA	PFNA	PFHxS	PFOS	PFOA	PFNA	PFHxS	
Nelson et al. [30] (sampling period 2003–2006)	US	Adolescents and adults (>12 y)	3953	$0–19,999	16.5	3.4	0.9	1.7	**−19.3(−24.6, −13.8)**	**−15.9(−22.5, −8.7)**	**−17.8(−26.5, −8.0)**	**−16.1(−24.1, −7.3)**	NHANES (2003–2006); Multivariable linear regression model adjusted for NHANES cycle, age, gender, race/ethnicity, creatinine.
				$20,000–44,999	17.9	3.7	0.9	1.8	**−11.6(−17.1, −5.8)**	**−10.1(−15.2, −4.6)**	**−11.3(−18.4, −3.5)**	**−11.0(−19.4, −1.7)**	
				$45,000–74,999	18.5	4	1	1.8	−5.4(−11.8, 2.4)	−1.3(−6.5, 4.2)	−6.6(−13.5, 0.8)	−5.3(−15.1, 5.6)	
				≥$75,000	19.8	4.2	1.1	2	Ref	Ref	Ref	Ref	
Brantsæter et al. [35] (sampling period 2003–2004)	Norway	Pregnant women (<25 y to >35 y)	487	Both < 300,000 NOK	12.5	2.16	0.36	0.59		Ref			Maternal education and household income both reflect socio-economic status and were not selected in the same multiple linear regression model.
				One ≥ 300,000 NOK	12.8	1.99	0.38	0.57		4.7(−2.6, 12.6)			
				Both ≥300,000 NOK	13.3	2.41	0.44	0.67		**12.6(3.6, 22.3)**			
Kato et al. [57] (sampling period 2003–2006)	US (Cincinnati)	Pregnant women (≥18 y)	180	<$20,000	9.44	4.1	0.64	0.84	**−37(−51, −19)**	**−30(−46, −10)**	**−23(−36, −7)**	**−51(−65, −31)**	Univariate linear regression model.
				$20,000–40,000	13.29	5.35	0.84	1.4	−11(−29, 13)	−9(−28, 15)	2(−14, 21)	−18(−41, 12)	
				$40,000–80,000	13.98	5.69	0.86	1.72	−6(−23, 15)	−3(−21, 19)	4(−10, 21)	0(−24, 32)	
				>$80,000	14.87	5.89	0.83	1.71	Ref	Ref	Ref	Ref	
Sagiv et al. [31] (sampling period 1999–2002)	US (Boston)	Pregnant women (<20 y to >35 y)	1645	<$40,000	24.3	5.3	0.6	2.3	**−9.8(−18.9, 0.3)**	**−11.1(−19.1, −2.4)**	**−18.5(−26.5, −9.7)**	−0.7(−14.7, 15.7)	Fully adjusted multivariable linear regression model; adjusted for year, age, race/ethnicity, education, marital status, smoking, parity, breastfeeding, BMI, gestational age, albumin, GFR.
				$40,000–70,000	26.9	5.7	0.6	2.4	3.2(−3.8, 10.6)	−4.5(−10.3, 1.5)	**−10.4(−16.3, −4.1)**	**−9.4(−18.0, 0.1)**	
				>$70,000	24.9	5.7	0.7	2.6	Ref	Ref	Ref	Ref	
Colles et al. [48] (sampling period 2012–2015)	Belgium (Flanders)	Adults (50–65 y)	168	≤€1250	6.348		0.729		Ref	Not included in model ^a^	Ref	Not included in model	Income is equivalent income ^b^: household income corrected for the number of persons in the household. Stepwise multiple linear regression model with age, BMI and gender forced into model.
				€1250–1600	6.066		0.702		−6(−29, 23)		−6(−27, 22)		
				€1600–2000	9.077		1.056		**34(5, 70)**		**38(9, 74)**		
				>€2000	8.792		0.975		**29(2, 63)**		21(−3, 51)		

Significant (*p* < 0.05) percent change indicated in bold; BMI: body mass index; GFR: glomerular filtration rate; GM: geometric mean; NOK: Norwegian Krone; PFOS: perfluorooctanesulfonic acid; PFOA: perfluorooctanoic acid; PFNA: perfluorononanoic acid; PFHxS: perfluorohexane sulfonate; Ref: reference; US: United States. In the column, % changes per income category from regression models, the 95% CI is given between brackets. ^a^: only variables for which the correlation was significant (*p* < 0.05) were included in the multiple linear regression model. ^b^: changes in PFAS concentration per equivalent household income are slightly stronger associated than for not corrected household income [30].

**Table 3:** Estimated Belgian median food consumption (g/day) in adults by education for major food categories. Data from De Ridder et al. [75].

Nr.	Educational Level	Potatoes and Potato Products	Fish, Fish Preparations, Shellfish	Meat & Meat Preparations	Vegetables	Fruit	Eggs
Group 1	ISCED0-2	44	25	131	152	71	8
Group 2	ISCED3-6	43	26	125	172	101	7
Group 3	ISCED7-8	36	29	107	205	115	8

ISCED: International Standard Classification of Education.

**Table 4:** Estimate on PFOS intake (ng/day) in Belgian adults through diet stratified by education.

Nr.	Educational Level	Unit	Potatoes and Potato Products	Fish and Seafood		Meat & Meat Preparations	Vegetables	Fruit	Eggs	Total
				Fish (Fresh and Marine)	Seafood					
Group 1	ISCED0-2	ng PFOS/day		4.4	5.1	5.6	0.5	6.9	0.5	23
Group 2	ISCED3-6		-	4.7	5.4	5.3	0.6	9.9	0.4	26
Group 3	ISCED7-8			5.2	6.0	4.6	0.7	11.3	0.5	28
	Percent change between group 1 and group 3 ^a^	%	-	+17	+17	−18	+34	+63	<1	+22
	Average contribution to total intake in percent	%	-	18	21	20	2	36	2	

ISCED: International Standard Classification of Education. ^a^: lowest education as reference.
